# Promoting Dental Health Through Teledentistry: Assessing Awareness and Attitudes in Saudi Arabia

**DOI:** 10.7759/cureus.55805

**Published:** 2024-03-08

**Authors:** Anas Alhur, Faisal Al Shahrani, Khlood Alasiri, Nourah Almutairi, Sarrah Almadi, Sami Alfazae, Mohammed Alqahtani, Mashael Aljehani, Mohammed Alqarni, Abdullah Al Qahtani, Fahad Alzahrani, Bandar Almaymuni, Rahaif Al Qobti

**Affiliations:** 1 Health Informatics, University of Hail College of Public Health and Health Informatics, Hail, SAU; 2 Public Health, Armed Forces Hospital Southern Region, Abha, SAU; 3 Dentistry, King Fahad Military Medical Complex, Dhahran, SAU; 4 Dentistry, Prince Sultan Military Medical City, Riyadh, SAU; 5 Dentistry, Ministry of Health, Riyadh, SAU

**Keywords:** teledentistry, attitudes, perceptions, awareness, assess

## Abstract

Introduction

Teledentistry has emerged as a promising solution to enhance dental healthcare accessibility and quality. Understanding public awareness and attitudes toward teledentistry is crucial for its successful implementation, especially in regions like Saudi Arabia where digital health initiatives are rapidly expanding. This study aims to assess the level of awareness, attitudes, and perceptions toward teledentistry among the Saudi Arabian population, as well as examine the demographic factors influencing its acceptance.

Methods

A cross-sectional survey was conducted with 474 participants, employing a structured questionnaire to collect data on demographics, awareness, knowledge, perceptions, and attitudes toward teledentistry. Statistical analyses, including correlation and chi-square tests, were performed to analyze the data.

Results

The majority of respondents (64%) reported awareness of teledentistry, primarily through the Internet (44.5%) and healthcare providers (36.3%). The average self-assessed knowledge score was 3.04 out of 5, indicating moderate familiarity with teledentistry concepts. Attitudes toward teledentistry were generally positive, with 54% expressing belief in its effectiveness. However, a significant portion of the sample showed reluctance toward using teledentistry for routine dental check-ups, with only 45.1% showing willingness. High technological accessibility was reported, yet 55.9% of participants preferred traditional in-person consultations. Correlation analysis revealed a moderate positive relationship between knowledge and belief in effectiveness (r = 0.21), but a negligible correlation with willingness to use teledentistry (r = 0.016). A strong positive correlation was found between belief in effectiveness and willingness for routine check-ups (r = 0.673). Educational level significantly influenced teledentistry acceptance, with higher education correlating with greater receptiveness.

Conclusion

While there is a moderate level of awareness and a generally positive attitude toward teledentistry among the Saudi population, hesitance remains regarding its use for routine care. The findings highlight the need for educational initiatives to improve knowledge and perceptions of teledentistry, potentially enhancing its acceptance and integration into the healthcare system.

## Introduction

The integration of telecommunications technology with dental services, known as teledentistry, is at the forefront of revolutionizing dental healthcare accessibility and quality worldwide. This innovation is particularly pertinent to regions like Saudi Arabia, where the quest to enhance oral health services is paramount [1]. Teledentistry stands out as a beacon of hope, offering the potential to bridge specialized dental care to the most remote and underserved areas, thereby promising a significant transformation in the healthcare landscape of the Kingdom [2,3].

However, the journey toward the widespread adoption of teledentistry in Saudi Arabia is fraught with challenges. These include varied levels of awareness and acceptance among the populace and dental professionals alike. Although some studies underscore the efficacy of digital platforms in dental health education and the promising role of teledentistry in clinical settings [4,5], concerns about diagnostic accuracy, cost implications, and data security loom large [6,7].

In light of these considerations, this study embarks on a critical assessment of the current awareness and attitudes toward teledentistry within the Saudi demographic. It aims to unearth the principal barriers and enablers to its adoption, thereby furnishing healthcare policymakers and dental practitioners with actionable insights. The ultimate ambition of this research is to pave the way for a strategic embrace of teledentistry in Saudi Arabia, aiming to elevate the standards of dental healthcare outcomes and accessibility [8].

## Materials and methods

This study employed a cross-sectional survey design to assess awareness, attitudes, and perceptions toward teledentistry among the Saudi Arabian population. The methodology was carefully crafted to ensure robustness, reliability, and relevance to the research objectives.

Sampling method

The study utilized stratified random sampling to ensure a representative sample of the adult population in Saudi Arabia. The population was stratified based on age, gender, educational level, and geographical location, mirroring the national demographic distribution as reported by the Saudi General Authority for Statistics. Within each stratum, participants were randomly selected to participate in the survey, aiming for a balanced representation across different demographic groups. The sample size was calculated using Cochran's formula, targeting a confidence level of 95% and a margin of error of 5%, resulting in a minimum required sample size of 474 respondents.

Questionnaire design

The questionnaire was developed based on a comprehensive review of the literature and existing validated instruments in the field of teledentistry and digital health acceptance. The questionnaire underwent a rigorous validation process, including expert review by a panel of dental professionals and digital health researchers to ensure content validity. A pilot test was conducted with a small subset of the target population (n=30) to assess the clarity, relevance, and reliability of the questionnaire items. Feedback from the pilot test was used to refine the questionnaire, resulting in a final instrument comprising three sections: (1) demographic information, (2) awareness and knowledge of teledentistry, and (3) attitudes and perceptions toward teledentistry (Appendices). The questions were a mix of multiple-choice, Likert scale, and open-ended responses to capture a broad spectrum of data.

Data collection

Data collection was exclusively conducted online over a three-month period to leverage the widespread use of digital platforms and ensure a broad reach across various demographics in Saudi Arabia. The online questionnaire was strategically disseminated through a variety of channels to enhance participation rates and diversity among respondents. These channels included targeted social media campaigns on platforms such as Twitter, Facebook, and LinkedIn, professional networks relevant to dental healthcare, and email lists compiled from educational and healthcare institutions.

Statistical analysis

Data were analyzed using SPSS version 25 (IBM Corp., Armonk, NY, USA). Descriptive statistics were used to summarize demographic information and the main study variables. Chi-square tests and independent t-tests were conducted to examine differences in awareness, attitudes, and perceptions toward teledentistry across different demographic groups. Pearson correlation coefficients were calculated to explore the relationships between awareness, attitudes, and willingness to use teledentistry. Advanced statistical techniques, including multiple regression analysis, were employed to identify significant predictors of teledentistry acceptance among the Saudi population.

Ethical considerations

Ethical approval for this study was obtained from the Ethical Approval Committee of the Research Department at Hail Health Cluster, with approval number 2024-52. Informed consent was obtained from all participants, who were informed about the study's purpose, procedures, and their rights, including confidentiality and the option to withdraw at any time without penalty. Data privacy was strictly maintained to protect participant information.

## Results

The study aimed to explore awareness and attitudes toward teledentistry within the Saudi Arabian population. A cross-sectional survey was conducted, garnering responses from 474 participants, as shown in Table 1. The demographic distribution included 297 males (62.7%, n=297) and 177 females (37.3%, n=177), with the age group of 25-34 years being the most represented (46.0%, n=218). A significant portion of respondents held a Bachelor's degree (64.3%, n=305), indicating a well-educated sample.

**Table 1:** Participant demographics

Category	Frequency (n)	Percent (%)
Gender		
Male	297	62.70%
Female	177	37.30%
Age		
Less than 18	36	7.60%
18 – 24	135	28.50%
25 – 34	218	46.00%
35 – 44	43	9.10%
45 – 54	42	8.90%
Education Level		
Below High School	3	0.60%
High School Graduate	3	0.60%
Some College/Vocational	37	7.80%
Bachelor’s Degree	305	64.30%
Postgraduate Degree	126	26.60%

The demographic data highlight a young and educated respondent pool, potentially indicative of greater receptiveness to new technologies like teledentistry.

A notable 64% (n=303) of the surveyed population reported prior awareness of teledentistry, primarily through the Internet (44.5%, n=211) and healthcare providers (36.3%, n=172). When asked to self-assess their knowledge of teledentistry, the average score was 3.04 (on a 5-point scale), with a standard deviation of 1.35, reflecting a moderate level of understanding (Table 2).

**Table 2:** Awareness and sources of information

Category	Subcategory	Percent (%)	Frequency (n)
Prior Knowledge of Teledentistry	Yes	64%	303
Source of Information			
Healthcare Provider		36.30%	172
Internet		44.50%	211
TV/Radio		0.60%	3
Friends/Family		16%	76

In assessing self-reported knowledge levels regarding teledentistry among the study participants, the data revealed a distribution across a 1-5 scale, where 1 represented 'Not Knowledgeable' and 5 denoted 'Highly Knowledgeable.' Out of the respondents, 134 (28.3%, n=134) rated themselves as 'Not Knowledgeable' about teledentistry, as demonstrated below in (Table 3). A majority of 276 participants (58.2%, n=276) considered themselves 'Moderately Knowledgeable,' falling within the 2-4 range on the scale. Meanwhile, 61 respondents (12.9%, n=61) felt confident enough to rate themselves as 'Highly Knowledgeable,' indicating a higher level of familiarity or understanding of teledentistry. The overall mean score for teledentistry knowledge among the participants was calculated at 3.04, with a standard deviation of 1.35, suggesting a moderate level of knowledge with some variability in the self-assessment responses.

**Table 3:** Self-assessment of teledentistry knowledge

Knowledge Level (1-5 Scale)	Frequency (n)	Percent (%)
1 (Not Knowledgeable)	134	28.30%
2-4 (Moderately Knowledgeable)	276	58.20%
5 (Highly Knowledgeable)	61	12.90%

The perceptions and attitudes toward teledentistry among participants were varied. For the belief in the effectiveness of teledentistry, 23.2% (n=110) strongly agreed and 30.6% (n=145) agreed, indicating a positive outlook by over half of the respondents. However, 30.6% (n=145) remained neutral, and a smaller fraction expressed disagreement (9.9%, n=47) or strong disagreement (5.1%, n=24). In terms of willingness to use teledentistry for routine dental check-ups, 35.2% (n=167) were likely willing, and 9.9% (n=47) were definitely willing, showing considerable interest in adopting teledentistry. On the other hand, 23.2% (n=110) were definitely unwilling, 12.2% (n=58) were unlikely willing, and 18.8% (n=89) remained undecided, reflecting some hesitance or reservations about its use; see Table 4 for more information.

**Table 4:** Perceptions and attitudes toward teledentistry

Description	Frequency (n)	Percent (%)
Belief in Effectiveness		
Strongly Agree	110	23.20%
Agree	145	30.60%
Neutral	145	30.60%
Disagree	47	9.90%
Strongly Disagree	24	5.10%
Willingness for Routine Check-Ups		
Definitely Willing	47	9.90%
Likely Willing	167	35.20%
Undecided	89	18.80%
Unlikely Willing	58	12.20%
Definitely Unwilling	110	23.20%

The accessibility to teledentistry was notably high among participants, with 98.1% (n=465) having access to reliable internet and 99.4% (n=471) possessing the necessary technology, see Table 5. Despite this high accessibility, preferences were divided when it came to the type of dental consultation. About 43.5% (n=206) of the participants preferred teledentistry while a slightly larger group (55.9%; n=265) still favored traditional in-person visits.

**Table 5:** Accessibility and preferences for teledentistry

Category	Frequency (n)	Percent (%)
Access to Reliable Internet	465	98.10%
Access to Necessary Technology	471	99.40%
Preference for Consultation Type		
Prefer Teledentistry	206	43.50%
Prefer Traditional Visits	265	55.90%

**Figure 1:**
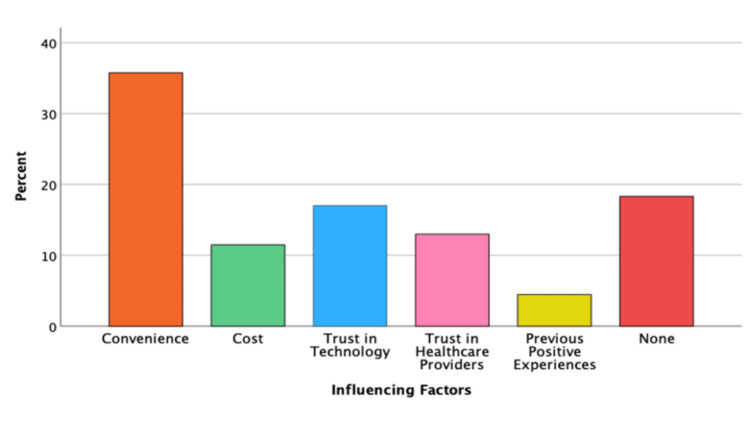
Factors influencing the utilization of teledentistry

The bar graph presented below indicates that 'Convenience' emerged as the predominant factor influencing teledentistry adoption, accounting for 37%. This is followed by 'non-category' factors at 18%, 'Trust in Technology' at 16%, 'Trust in Healthcare Providers', 'Cost,' and finally, 'Various Positive Experiences', as demonstrated in (Figure 1).

Our analysis further explored the relationships between participants' self-assessed knowledge of teledentistry, their belief in its effectiveness, and their willingness to use it for routine dental check-ups. The findings, summarized in Table 6, show varying degrees of correlation among these variables.

**Table 6:** Correlation analysis summary

Variables	Correlation Coefficient	Interpretation	n
Self-Assessment Knowledge & Belief in Effectiveness	0.21	Moderate positive correlation	474
Self-Assessment Knowledge & Willingness for Routine Check-Ups	0.016	Very low correlation	474
Belief in Effectiveness & Willingness for Routine Check-Ups	0.673	Strong positive correlation	474

A moderate positive correlation (r = 0.21) was observed between participants' self-assessed knowledge (n=474) and their belief in the effectiveness of teledentistry. This indicates that as individuals' self-reported knowledge about teledentistry increases, so does their belief in its effectiveness. The strength of this correlation suggests that educational interventions aimed at improving knowledge about teledentistry could positively impact perceptions of its efficacy.

However, the correlation between self-assessed knowledge (n=474) and willingness to use teledentistry for routine check-ups was found to be very low (r = 0.016). This implies that while knowledge of teledentistry may influence beliefs about its effectiveness, it does not necessarily lead to a higher willingness to use these services for regular dental care.

In contrast, a strong positive correlation (r = 0.673) was found between the belief in the effectiveness of teledentistry (n=474) and willingness to use it for routine check-ups. This significant finding suggests that the perception of teledentistry as an effective form of dental care is a powerful predictor of individuals' willingness to use it. It highlights the importance of promoting the proven benefits and effectiveness of teledentistry to increase user adoption rates.

In examining demographic factors influencing the acceptance of teledentistry, our statistical analysis identified distinct patterns. The chi-square test, used to assess the strength of association between two categorical variables, analyzed the relationship between participants' gender (n=474) and education level (n=474) with their willingness to engage in teledentistry for routine dental check-ups.

The chi-square test for gender yielded a value of 6.61 with a p-value of 0.158, indicating no statistically significant difference between males (n=297) and females (n=177) in their willingness to use teledentistry within our sample population. This suggests that gender may not be a critical factor in the likelihood of adopting teledentistry services for routine dental care.

Conversely, the association between education level and willingness to use teledentistry was statistically significant, with a chi-square value of 37.69 and a p-value of 0.0017. This finding demonstrates that educational background significantly influences the acceptance of teledentistry, with individuals of higher educational attainment more likely to be receptive to using teledentistry services. This may reflect varying levels of familiarity with or trust in digital health solutions among different educational groups, as seen in Table 7.

**Table 7:** Chi-square test summary

Association Between	Chi-Square Value	p-value	Significant at α=0.05	n
Gender & Willingness for Routine Check-Ups	6.61	0.158	No	474
Education Level & Willingness for Routine Check-Ups	37.69	0.0017	Yes	474

## Discussion

The incorporation of digital technologies in healthcare, as evidenced by Saudi Arabia's evolving practices, reflects a broader trend toward enhanced technological integration in healthcare systems worldwide [9-13]. This investigation into the perceptions and receptivity toward teledentistry among the Saudi populace not only aligns with existing scholarship but also unveils unique insights pertinent to the socio-cultural and technological context of Saudi Arabia.

The findings indicate a nascent awareness and understanding of teledentistry among participants, suggesting an emerging recognition of its potential to transform dental healthcare delivery. This aligns with Alkhalifah and Al-Nasser's (2020) findings on the growing importance of digital platforms for health information dissemination in Saudi Arabia [14]. Moreover, the identified role of healthcare professionals in information dissemination highlights the necessity for increased digital literacy and engagement within the medical community to foster a comprehensive understanding and acceptance of teledentistry [15].

Despite the generally positive global perceptions of teledentistry's efficacy [16], our study reveals a notable reluctance among the Saudi population to adopt teledentistry for routine dental consultations. This reluctance, attributed to concerns over the impersonal nature of virtual consultations and potential privacy breaches, echoes the findings of Estai et al. (2016), who noted similar patient reservations toward remote healthcare services [17]. Addressing these concerns through robust privacy measures and enhancing the personalization of teledentistry interactions could mitigate hesitancy and bolster user trust and acceptance.

Our study also highlights Saudi Arabia's significant investment in digital infrastructure, suggesting a strong foundation for the adoption of teledentistry [18]. However, the preference for traditional in-person consultations, despite technological readiness, underscores the intrinsic value of direct patient-provider interactions in healthcare, advocating for a balanced approach to digital and conventional care [19,20]. This suggests that a hybrid model integrating teledentistry with traditional care could offer a more patient-centered solution, reflecting the nuanced preferences of the Saudi population.

Implications for practice and policy

These insights carry profound implications for healthcare practice and policy formulation in Saudi Arabia. There is a clear imperative for targeted educational initiatives aimed at enhancing the populace's understanding of teledentistry and addressing prevalent concerns. Such campaigns should not only demystify teledentistry but also highlight the measures in place to safeguard patient privacy and the efforts to maintain the personal touch in patient-provider interactions.

Furthermore, the readiness of the Saudi population for digital health solutions, juxtaposed with their inclination toward traditional care modalities, advocates for the development of a hybrid healthcare model. This model should seamlessly integrate teledentistry services with conventional dental care, tailored to accommodate individual patient preferences and clinical needs. This approach could serve as a blueprint for patient-centered care in the digital age, ensuring that technological advancements in healthcare augment rather than replace the human elements of care provision.

Future directions

Future research should focus on longitudinal studies to track the evolution of attitudes toward teledentistry over time, particularly as technological advancements and policy interventions unfold. Additionally, qualitative studies exploring the subjective experiences of both patients and providers with teledentistry could offer deeper insights into the barriers and facilitators of its adoption, informing more nuanced strategies for its integration into mainstream dental care.

Limitations

This study, while providing valuable insights into the awareness and attitudes toward teledentistry in Saudi Arabia, is subject to certain limitations that warrant consideration. Acknowledging these limitations is crucial for a balanced interpretation of the findings and for situating the study within the broader context of digital health research.

Sample representativeness

One of the primary limitations pertains to the representativeness of the sample. Despite efforts to reach a diverse population through online distribution channels, the inherent nature of online surveys may lead to a self-selection bias. This bias occurs as individuals with higher digital literacy and a predisposition toward digital platforms, including social media and email, are more likely to participate. Consequently, the sample may overrepresent individuals who are already inclined toward technology, potentially skewing the results toward a more favorable view of teledentistry.

Moreover, the online-only data collection approach may have limited access for certain demographic groups, particularly older adults, those in rural areas with limited Internet connectivity, and individuals with lower socioeconomic status who may have restricted access to digital devices. This limitation could affect the generalizability of the findings to the entire Saudi Arabian population, especially in understanding the attitudes of less digitally connected individuals toward teledentistry.

Potential biases

In addition to sample representativeness, potential biases inherent in the study design must be acknowledged. The reliance on self-reported data introduces the possibility of response bias, where participants might provide socially desirable answers or may not accurately recall their experiences or perceptions. This bias could lead to an overestimation or underestimation of the actual awareness and positive attitudes towards teledentistry.

Another potential bias arises from the language used in the survey. Although the questionnaire was made available in Arabic to accommodate the primary language of the target population, nuances in language and the interpretation of questions could still influence how participants understand and respond to the survey items. This linguistic bias may impact the accuracy of the data collected, particularly for nuanced concepts related to digital health.

Interpretation and generalizability

These limitations highlight the need for a cautious interpretation of the study's findings. While the results provide meaningful insights into the current landscape of teledentistry awareness and attitudes in Saudi Arabia, they should be considered within the context of the mentioned limitations. The findings may not fully capture the perspectives of all demographic groups within the country, particularly those less engaged with digital platforms.

## Conclusions

This study reveals a moderate awareness and generally positive perceptions of teledentistry in Saudi Arabia, alongside a strong preference for traditional dental consultations. Despite the widespread availability of technology, the adoption of teledentistry is hindered by several challenges, highlighting the need for targeted educational efforts to increase acceptance and understanding.

The findings suggest that a patient-centered approach, integrating both digital and conventional dental services, could improve teledentistry's acceptance and usage. These insights are crucial for informing future healthcare policies and the strategic implementation of teledentistry, with the potential to significantly enhance dental healthcare accessibility and efficiency in the region.

## Figures and Tables

**Figure 2:**
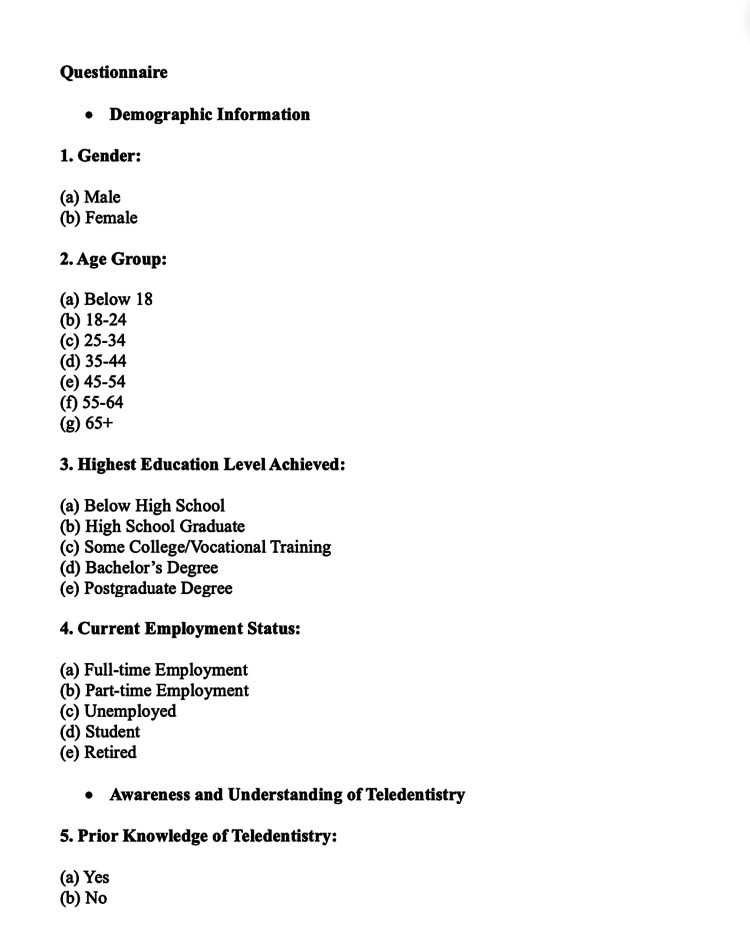
Questionnaire A

**Figure 3:**
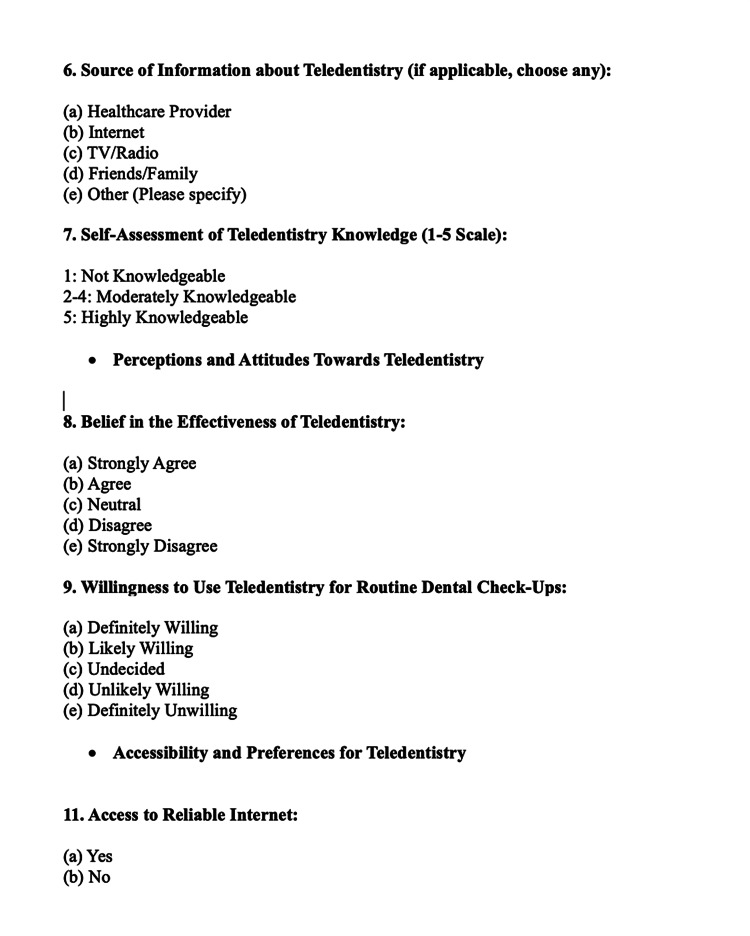
Questionnaire B

**Figure 4:**
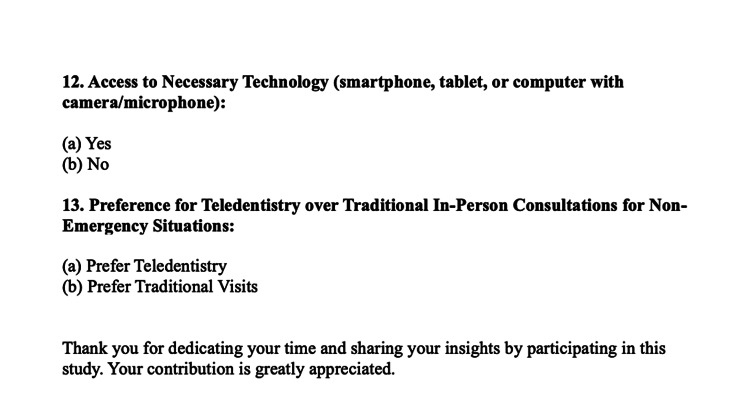
Questionnaire C
